# Development of a Questionnaire to Measure Public Perceptions of the Role of Community Pharmacy in Public Health (*PubPharmQ*)

**DOI:** 10.3390/pharmacy11050141

**Published:** 2023-09-08

**Authors:** Delyth H. James, Rose Rapado, Sarah L. Brown, Joanne Kember, Karen L. Hodson, Amie-Louise Prior

**Affiliations:** 1 Department of Applied Psychology, Cardiff School of Sport & Health Sciences, Cardiff Metropolitan University, Llandaff Campus, Western Avenue, Cardiff CF5 2YB, Wales, UK; roserapado@hotmail.co.uk (R.R.); sabrown@cardiffmet.ac.uk (S.L.B.); aprior@cardiffmet.ac.uk (A.-L.P.); 2 Betsi Cadwaladr University Health Board, Bangor LL57 2PW, Wales, UK; jojoreld@hotmail.co.uk; 3 School of Pharmacy & Pharmaceutical Sciences, Cardiff University, Redwood Building, King Edward VIIth Avenue, Cardiff CF103NB, Wales, UK; hodsonkl@cf.ac.uk

**Keywords:** public perceptions, public attitudes, service-user perspectives, community pharmacy, pharmacy services, public health, community pharmacy roles, questionnaire

## Abstract

Background: Community pharmacies are well placed to provide public-health interventions within primary care settings. This study aimed to establish the general public’s perceptions of community pharmacy-based public-health services in the UK by designing a structured questionnaire to assess the barriers and facilitators to optimizing this role. Methods: A standardized questionnaire was developed informed by the literature, additional semi-structured interviews, and synthesis of key findings with the authors’ previous research based on data generated from eight focus groups. The original 42-item questionnaire was distributed online from May to June 2021 via social media platforms to capture the views of non-regular pharmacy users. Following exploratory factor analysis, and Cronbach’s alpha analysis, total Likert scale response scores were calculated. Results: Of the 306 responders, 76.8% were female with a mean age of 34.5 years (SD = 15.09). The most prevalent pharmacy use reported was 1–2 times a year (28.1%). Exploratory factor analysis revealed four scales: *Expertise*, *Role in Public Health*, *Privacy*, and *Relationship* (18 items) with acceptable internal consistency and good face and content validity. Awareness of well-established pharmacy services was high; however, responders demonstrated poor awareness of public-health-related services and low recognition of pharmacy expertise for this role. A lack of an established relationship with community pharmacies and privacy concerns were also perceived barriers. Conclusions: Based on these findings, considerable effort is needed to increase public awareness and address these concerns if strategic plans to utilize community pharmacy in the delivery of public-health policy are to be successful. The *PubPharmQ* provides a novel, structured questionnaire to measure the public’s perceptions of community pharmacy’s role in public health.

## 1. Introduction

The World Health Organization (WHO) acknowledges that community pharmacies are one of the most accessible ways for patients and the public to gain healthcare advice [1]. Community pharmacies in the United Kingdom (UK) are well placed to offer public-health interventions, as they are based in the heart of communities, including deprived areas [2,3]. Examples of public-health services include those which reduce the prevalence of high-risk behaviors and promote a healthy lifestyle such as smoking cessation and increasing vaccination rates [4]. Traditionally, community pharmacy funding models in the UK have been centered on the supply of medicines [4] however, although the contractual frameworks across the four devolved nations (England, Northern Ireland, Scotland, and Wales) all contain public-health services, there are substantial variations. Recent changes in health policies and the community pharmacy contractual framework in Wales have resulted in the implementation of further public-health-focused services to help protect the health of the nation [5].

Research relating to perceptions of the role of community pharmacies in public health has largely been aimed at service users who often already have experience of using pharmacies for dispensing their National Health Service (NHS) prescriptions [6,7,8,9,10,11]. A systematic review conducted in 2011 [8] investigated community pharmacists and service-user attitudes toward the role of pharmacies as providers of public-health advice. They found that service users felt they rarely received public-health services from community pharmacies and were unsure whether pharmacy staff had the expertise to perform such a role. However, those who had experience receiving public-health advice from a pharmacy were generally satisfied with the service.

Key to the successful implementation of any policy development for the expansion of community pharmacy services and public-health roles is to identify the barriers and facilitators from the general public’s perspective who may not have any previous contact with a community pharmacy [12]. In 2010, Krska & Morecroft [13] explored the views of the general public about the role of pharmacy in providing public-health advice, but many respondents said they would not use the services due to issues around confidentiality, privacy, space, and “busyness”. In 2012, Gidman et al. [14] published their research which explored public experiences and opinions of pharmacy services in Scotland. The study found that many members of the public preferred to access their General Practitioner (GP) for public-health advice, suggesting that improved communication and information sharing between the GP and the pharmacy is essential to support the development of NHS pharmacy-led public-health services. Two further small-scale qualitative studies explored how the general public perceives community pharmacies in Scotland and Wales, respectively [15,16], where both uncovered a general view that the focus of community pharmacies was to supply medicines rather than having a role in providing public-health-related services. Community pharmacies were generally seen as the first port of call when it came to medicine or prescription-related inquiries, but there was a distinct lack of awareness of the public-health role. However, when study participants were informed of such services, there was much positive feedback, implying a high level of potential acceptance for these roles [16] Community pharmacists in general were deemed as being professional; however, one barrier identified was the type of community pharmacy in which they worked. For example, supermarket pharmacies (i.e., pharmacies owned by or located within a supermarket) were viewed less favorably than the smaller pharmacies, which were perceived as being less commercially driven [16]. This view was supported by Saramunee and colleagues’ findings that a pharmacy near home or a doctor’s surgery and long opening hours were preferred for the provision of public-health services [17] Since then, further research has focused on the public’s response to the role of community pharmacy in specific areas of public-health such as diabetes prevention, health and wellbeing, the COVID-19 pandemic and children’s health interventions [18,19,20,21].

A poll undertaken by IPSOS Mori in 2015 [22] found that as many as 65% of respondents visited a community pharmacy to pick up a prescription; however, when it came to receiving advice about a health problem, it reduced to 9%, and this figure fell further to 3% for visiting a pharmacy for public-health services. These data indicate that the skills and expertise of community pharmacy teams in the delivery of public-health services are not being utilized. In February 2023, the UK Office for National Statistics found that 19% of adults surveyed in the UK needed to contact their GP but chose not to, due to perceptions of long waiting times, accessibility of appointments, and high burden on the NHS [23]. Of these, 14% chose to consult a pharmacist instead of their GP. However, this percentage is much less than that for individuals who reported attempting to manage the condition themselves (57%) or seeking advice on the Internet (22%) [23].

In summary, many members of the general public may not have had regular exposure to community pharmacy services. They are therefore unlikely to use a pharmacy for public-health-related services if they are unaware of their existence or do not understand where and how those services are delivered. The focus of this research was to investigate whether there may be perceptions embedded in the general public’s mindset, which prevent them from using community pharmacies for public-health-related services. It would also seem that public perceptions in the UK have not changed over the last decade or more but there is a need to capture this information regularly. Building on previous research [6,7,8,9,10,11,13,14,15,16,17] in this area, a robust measure for capturing public opinion of community pharmacy’s role in public health is needed.

The aim of this study was therefore two-fold; first, to design and test a questionnaire for measuring the public’s perceptions of community pharmacy-based public-health services; second, to establish the nature and frequency of the barriers and facilitators to the uptake of public-health services in community pharmacies.

The objectives were:

1. To design a questionnaire to capture public perceptions of community pharmacy’s role in delivering public-health services.
2. To quantify the degree to which the public accepts using community pharmacies for engagement in public-health services.
3. To identify the barriers and facilitators to public engagement with pharmacy-based delivery of public-health services.

## 2. Materials and Methods

### 2.1. Overview of Study

A two-staged approach was adopted using qualitative and quantitative methodologies to capture the public’s perceptions of the role of community pharmacy from a public-health perspective. Stage 1 comprised a review of the literature and semi-structured interviews to augment qualitative, focus group research previously conducted by the authors [16]. Stage 2 involved the development and testing of a structured questionnaire which consisted of items generated from Stage 1 [24] The questionnaire was distributed online via social media to capture the general public’s views about reasons for visiting a community pharmacy and their role in delivering public-health services.

### 2.2. Ethics

Research ethics approval was gained from Cardiff Metropolitan University, School of Sport and Health Sciences Ethics Committee. Reference UG-1613 (Stage 1); PG-4163 (Stage 2). Interview participants for Stage 1 were recruited following the provision of written informed consent. Data from interviewees were anonymized to ensure that neither the participant nor the specific pharmacy were identifiable. For Stage 2—informed consent was obtained on the first page for all participants completing the online survey.

### 2.3. Stage 1

#### 2.3.1. Participants, Sampling, Setting and Recruitment

Individuals from one university in Southeast Wales (representing members of the general public with little or no experience of using community pharmacies) were invited to take part in one-to-one interviews. E-mail invitations were sent to undergraduate students studying BSc Psychology who were members of the Participant Panel for research. Posters were also distributed in public areas on one of the University’s campuses. The inclusion criteria were over 18 years of age and not receiving regular prescribed medication from a pharmacy.

#### 2.3.2. Interview Schedule, Data Collection and Analysis

A semi-structured interview schedule was developed (Supplementary Material S1), adapted from the focus group topic guide used in the authors’ previous research [16] and modified to incorporate the updated literature in this area [9,10,11,17,18,19,20] Key areas addressed were (1) *Professionalism*, (2) *Commercialism*, and (3) *Role of community pharmacies*. Open-ended questions were used to explore participants’ views about these themes with a list of prompts to provide further explanation where appropriate. Inclusion criteria were over 18 years of age and from any part of the UK. Demographic details (age, gender, ethnicity) were collected at the end of the interviews. Interviews were conducted face to face from January to March 2020, recorded and transcribed *verbatim* for inductive thematic analysis [25]. Deductive analysis was only applied to data relating to perceptions of the type of pharmacy. All themes generated from these semi-structured interviews and previously reported focus group themes were collated and used to inform Stage 2.

### 2.4. Stage 2

#### 2.4.1. Questionnaire Development and Piloting

The themes derived from the analysis of interview data in Stage 1 were compared with themes developed from focus group data from our previous research [16] to identify areas of alignment and used to design the questionnaire. Issues identified in the published literature in this area also informed the questionnaire statements. Questionnaire items were generated for each theme and adapted from *verbatim* quotes where appropriate [26]. Five-point Likert scale responses were used for all attitudinal questions and some items were negatively worded to allow testing for response reliability [27]. These were organized into five sections consisting of 42 items in total. Eight experts reviewed drafts of the questionnaire as part of the iterative process of development, comprising: three pharmacists and one pharmacy technician practicing in community pharmacy; two pharmacists working in academia; and two health psychology academics with expertise in questionnaire design.

Other sections of the questionnaire collected demographic information including age, gender, location within the UK (First 4 digits of postcode), ethnicity, employment status, and whether they received regular prescription medication from their GP. Respondents were also asked to state the type of pharmacy (i.e., small multiple, large multiple, supermarket pharmacy, or independent pharmacy, with named examples provided for each) used in the past year for prescription and public-health needs, whether they had received a one-to-one consultation with pharmacy staff in a consultation room. They were also asked to indicate which type of pharmacy they preferred for different reasons, e.g., which was considered best for privacy, professionalism, and comfort when attending for public-health support (See Supplementary Material S3 for Version 1 of the questionnaire). NOTE: Community pharmacies in the UK can be independently owned, part of a small multiple (a chain of over five pharmacies) [28,29], a large multiple (over 100 chains e.g., Boots), or located within or part of a supermarket chain (e.g., Tesco’s) [30].

Questionnaire drafts were pre-tested with 11 individuals (from England and Wales) as a sense-check to ensure that the questionnaire worked as intended [31], five of whom were regular service users and the others had little or no experience of using community pharmacies. Each person was asked to complete the questionnaire either on paper or test its user-friendliness online and provide feedback on issues such as how long it took to complete and clarity of questions and instructions. Key changes included the substitution of ‘neither agree nor disagree’ for ‘uncertain’ as a response option, the addition of space for further comments, the inclusion of an example of a situation when a consultation room is used, and minor alteration to the wording of some questions specifically those relating to supermarket pharmacies.

#### 2.4.2. Participants, Sampling, Setting and Recruitment

The questionnaire was launched online and advertised online via social media platforms (LinkedIn™, Twitter™, Facebook™, and Instagram™) to recruit an opportunistic [32] self-selecting sample of participants. Inclusion criteria were over 18 years of age and resident in the UK. Social media was viewed as an appropriate way to recruit participants as it can be an accessible method of recruitment [33] to minimize researcher bias and potentially maximize distribution, particularly with some hard-to-reach populations [34].

#### 2.4.3. Data Collection

The questionnaire (Supplementary Material S3) was distributed using the online Qualtrics survey platform [35] during May and June 2021. Participants were able to view an information sheet that provided some background details about community pharmacy and public-health services. It was estimated to take between 10 and 15 min to complete based on the piloting feedback. Participants were able to withdraw from the study at any point by exiting the online survey and these respondents were not included in the analysis.

#### 2.4.4. Analysis

Data were imported to and Statistical Package for Social Sciences (SPSS) Version 25 [36] databases on a secure OneDrive account. Data were cleaned, by removing respondents who were not part of the study inclusion criteria—for example, non-UK residents (*n* = 3) and underage respondents (*n* = 1). All other cases were included in the analysis. There were no missing data since the survey was set up on Qualtrics in a way that prevented participants from moving on to the next question if any responses were omitted. Negatively worded question scores were reversed so that a high score was converted to a corresponding low score [37]. Exploratory factor analysis was chosen to decide if there were underlying dimensions of the set of variables [38] and an underlying structure to the pattern of correlations [39] allowing possible options for new scales to be established. There were sufficient responses (*n* = 306) for factor analysis to be appropriate as there were more participants than extracted factors [39]. The Kaiser Meyer-Olkin (KMO) measure of sampling adequacy (=0.913) and Bartlett’s test (x_2_ = 5871.876, df = 703, *p* < 0.001) was significant, meaning that factor analysis could proceed. A loading of 0.33 or above on the rotated components matrix was considered when grouping factors. This was based on the guidelines for identifying significant factor loadings for sample size (where *n* of 350 = 0.30; *n* of 250 = 0.35; *n* = 200 = 0.40 etc.) since our sample size of 306 was between 250 and 350 [40]. Following the original exploratory factor analysis distribution, using the varimax rotation method, Cronbach’s alpha analysis was conducted to test the reliability of the new scales to ensure good internal consistency (i.e., alpha value above 0.7) [41]. Total scores were calculated for each scale by summing the scores for each item.

Parametric tests were conducted on normally distributed data where frequency distributions of scale scores were explored. Linear relationships between variables were explored using Pearson correlation. The validity of scales was tested by exploring expected positive relationships between variables (e.g., *Relationship* and *Privacy* scale scores). Spearman’s correlation was conducted where data were not normally distributed (e.g., frequency of CP use and scale scores). A one-way ANOVA was conducted to examine differences in scale scores between UK regions and repeat prescription usage [41].

## 3. Results

### 3.1. Stage 1—Interviews

#### 3.1.1. Participants

A convenience sample of eight participants took part in the interviews, seven were undergraduate students and one member of staff. Seven self-identified as female and one as male. In terms of ethnicity, three participants were White, two mixed race, one Indian, one White/Asian, and one self-identified as non-White.

#### 3.1.2. Thematic Analysis

Inductive thematic analysis of the interview data conceptualized five themes. These were: Perceived Role, Public Awareness, Perceived Capability, Privacy Concerns, and Lack of Established Connection with a Pharmacy. Perception of the Type of Pharmacy was a sixth theme conceptualized from deductive thematic analysis. A thematic map and description of all six themes, including illustrative quotations are presented in Supplementary Material S2.

#### 3.1.3. Mapping of Interview and Focus Group Themes

Themes and sub-themes from the interviews in Stage 1 were aligned with themes from the previous focus group data [8] to allow comparison, and identification of similarities to inform the development of the questionnaire. These are presented in Table 1.

### 3.2. Stage 2—Questionnaire Development

Based on the six themes generated in Stage 1 and a mapping of these data to the five overarching themes developed in our earlier focus group study [16], items were produced to create the first version of the questionnaire (Supplementary Material S3). This consisted of 42 items categorized into five sections relating to the key areas of theme alignment (Table 1). These were: *Role in Public Health*, *Reasons for Visiting*, *Relationship*, *Communication*, and *Privacy*.

#### 3.2.1. Questionnaire Responses and Participant Characteristics

The total number of responses was 310 with 306 valid responses following the removal of four cases who did not meet the inclusion criteria. Demographic characteristics of participants are presented in Table 2.

The mean age of the participants was 34.5 years (SD = 15.09). Of these, 76.8% (*n* = 235) identified as female All valid responders lived in the UK, where most (61.1%, *n* = 187) were from England or (34.3%, *n* = 105) Wales. In terms of ethnicity, most participants were White (85.6%, *n* = 263), either full-time students (43.1%, *n* = 132) or in full-time employment (28.4%, *n* = 87). Nearly half of the participants said they received repeat prescriptions (49.7%, *n* = 152), and another 49.7% (*n* = 152) said they did not receive repeat prescriptions.

#### 3.2.2. Community Pharmacy Use

Table 3 shows the frequency of community pharmacy use over the previous year. Most participants stated that they visited their community pharmacy once or twice in the last year (*n* = 86; 28.1%), followed by three to four times in the past year (*n* = 62; 20.3%). Only 12.7% (*n* = 39) reported not using a pharmacy at all in the last year whereas 4.9% (*n* = 15) claimed to visit the community pharmacy 25 or more times in a year.

Most participants (57.8%, *n* = 177) stated that they had not used or offered to use a private consultation room when visiting a pharmacy for any purpose, with 39.9% (*n* = 122) reporting that they had used a consultation room and 2.3% (*n* = 7) not sure if they had used one or not.

#### 3.2.3. Type of Pharmacy Used

Table 4 presents responders’ experiences of the type of pharmacy used over the previous year for their prescription and public-health needs. The most frequently used type of pharmacy for prescription needs was small multiples (34%) and supermarket pharmacies (28.8%), whereas large multiples were the least frequently used (6.9%) for this purpose. In contrast, participants mostly used large multiples for their public-health needs (51.6%), followed by independent (43.1%) and small multiples (35.6%). Supermarket pharmacies were the least frequently used option (25.2%) for this purpose, while 23.5% had not visited any pharmacy for public-health advice in the past year.

#### 3.2.4. Type of Pharmacy Preference

Participants reported that independent pharmacies, large and small multiples were perceived to be the most professional (34%, 31.7%, and 28.8%, respectively) whereas supermarket pharmacies had the lowest score (6.9%). Participants stated that they would feel most comfortable going to large multiple pharmacies (39.9%) with independent (37.9%), small multiples (33%), and no preference (30.1%) also frequently reported. Comfort was reported least frequently for supermarket pharmacies, where only 11.4% felt most comfortable. No one type of pharmacy was reported as being most concerned with public-health matters, with most stating no preference (30.1%) for this and independent (25.2%), small multiple (27.1%), and large multiples (26.5%) had similar scores. However only 8.2% selected supermarkets as a preference for public-health matters. In terms of privacy, most participants felt that independent pharmacies had the most privacy (36.6%), followed by small (28.4%) and large (25.2%) multiples, whereas supermarket pharmacies had the lowest privacy score (6.2%) (Table 5).

#### 3.2.5. Exploratory Factor Analysis

Eight main factors with an Eigenvalue over 1 were initially extracted from the data (See Supplementary Material S4, Table S1). accounting for 61.93% of the variance in the data. However, four factors were retained which accounted for 40.63% of the variance in public perceptions of community pharmacy’s role in public health. The first factor (*Expertise*) accounted for 30.8% of the variance, the second (*Relationship*) 7.1%, the third (*Privacy*) 5.4%, and the fourth (*Role*) 5.1%. Decisions about the number of factors to retain in the questionnaire were based on the interpretation of the latent constructs [24]. The other four factors contained items that related to the same constructs as those retained and were therefore deemed to be unnecessary duplication.

The simplified rotated component matrix for Version 2 of the questionnaire is presented in Supplementary Material S4, Table S2. All items retained exceeded the factor loading cut-off value of 0.33 (ranging from 0.332 to 0.807). Supplementary Material S4, Table S3 outlines the original (Version 1) and corresponding items dependent on how they loaded together once rotated.

Version 2 of the questionnaire with four newly labeled scales consisting of 18 items is shown in Table 6. The four scales represented public perceptions of community pharmacy’s *Expertise* (3 items) and *Role in Public Health* (4 items) as well as capturing the *Relationship* (8 items) with the public and issues relating to concerns about *Privacy* (3 items). All subsequent analyses are based on the 18-item questionnaire.

#### 3.2.6. Reliability Testing and Descriptive Statistics

Table 7 presents the scale scores and Cronbach’s alpha values for each of the four scales of the questionnaire which all demonstrated a strong level of internal consistency with scores ranging from 0.745 to 0.862 indicating good internal reliability [14] for the four scales.

Frequency distributions of scale scores are presented in Supplementary Material S5. Results indicate that participants had a very low appreciation of the community pharmacy’s *Role* and *Expertise* in public health (only 12.7% and 7.8% above the scale midpoint scores, respectively). Scale scores for *Relationship* with community pharmacy (27.1% above midpoint score) also showed a low appreciation of the therapeutic relationship and over half reported that CPs offer sufficient *Privacy* (54.6% above the scale midpoint).

#### 3.2.7. Relationships between Scales

Table 8 presents the correlations between the four scale scores. As expected, strong statistically significant positive correlations were found between all four scales providing good support for the construct validity of the scales.

### 3.3. Relationships between Participant Characteristics and Scale Scores

There was a significant weak negative correlation between age and *Privacy* scores (r = −0.133, *N* = 306, *p* < 0.05) indicating that older people had more privacy concerns. There was a significant negative correlation between age and *Relationship* (r = −0.244, *N* = 306, *p* < 0.001) and *Role in Public-Health* scores (r = −0.145, *N* = 306, *p* < 0.05) indicating that younger people reported a better perceived relationship with the pharmacy team and of their role in public health. No significant correlation was found between age and *Expertise* scores. There was no significant effect of receiving repeat prescriptions on any of the total scale scores indicating that experience of using a pharmacy did not influence these perceptions.

**Figure 1:**
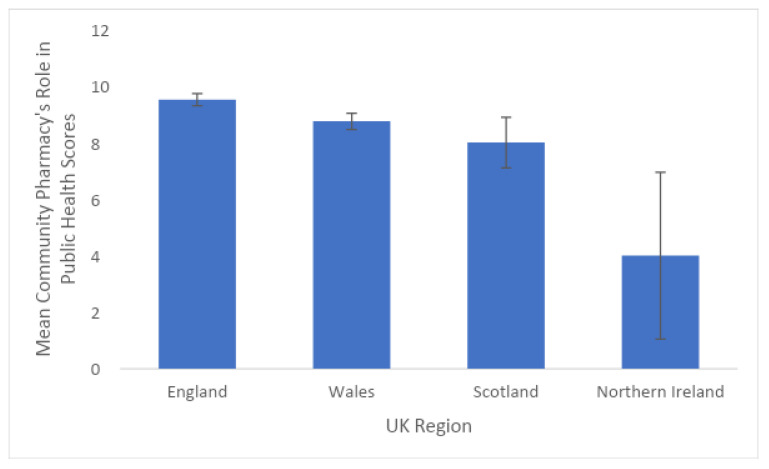
United Kingdom region by mean role score with standard error (*n* = 306).

There was no effect of the UK region on *Relationship*, *Expertise*, or *Privacy* scores. There was an effect of nation of the UK on perceived *Role in Public-Health* scores (f (4) = 2.411, *n* = 304, *p* < 0.05) where individuals in England scored the highest (9.519) and Northern Ireland scored the lowest with the largest amount of standard error (4.0, SE = 2.954; see Figure 1), (NOTE: this was based on one respondent only from Northern Ireland). Pairwise comparisons with a Tukey correction indicated a significant interaction between England and Wales (mean difference = 0.766, *p* < *0*.05) indicating a better overall perception of the community pharmacy’s role in public health in England (See Figure 1).

There was a significant negative correlation between the frequency of pharmacy use and *Relationship* scores (rs = −0.252, *N* = 306, *p* < 0.001). There was a significant weak negative correlation between the frequency of pharmacy use and *Role in Public Health* (rs = −0.142, *N* = 306, *p* < 0.05) and *Expertise* (rs = −0.190, *N* = 306, *p* < 0.05) scores, respectively. However, there was no correlation between the frequency of pharmacy use and *Privacy* scores.

## 4. Discussion

This study involved the design and testing of a questionnaire to quantify public perceptions of community pharmacies to determine the barriers and facilitators of using their services for public-health reasons. This was achieved through a mixed-methods approach, adopting a multi-staged study design. The application of exploratory factor analysis to the initial 42-item questionnaire yielded a short eighteen-item version of the questionnaire (*PubPharmQ*) for use in future studies to explore public perceptions of community pharmacy’s roles in delivering public-health services. A more concise questionnaire may improve participant engagement and retention, in future studies such as these, since individuals are more likely to complete it [42]. The new sub-scales and corresponding items, drawn from factor analysis and underpinned by qualitative research, demonstrated high internal consistency, good face, content, and construct validity, supporting its future use. The questionnaire allows public perceptions to be explored regarding awareness of the role of community pharmacies in public health, their expertise for this role, views about privacy, and the relationships in place for delivering public health services. The *PubPharmQ* can also be used to establish any potential changes in perceptions and acceptance of this role over time or measure changes in perceptions following an intervention to promote these services to the public. However, questionnaire design is an iterative process [27], so further work is needed to continue the psychometric testing of the *PubPharmQ*, to determine its utility for predicting pharmacy use and to discriminate between pharmacy and non-pharmacy users for public-health services. Aspects of the questionnaires’ validity and reliability will now be discussed in further detail.

### 4.1. Validity

Measures of validity are critical to consider during questionnaire design. Face validity determines whether the questionnaire appears sensible at face value [43] This was explored by piloting the questionnaire among individuals who had no experience of using a pharmacy as well as those who were familiar with the community pharmacy setting. Content validity ensures the questionnaire’s content is appropriate as judged by experts in the field [43]. In this study, content validity was embedded into the study design since the questionnaire items were derived from extensive qualitative research and informed by the published literature. Content validity was further ensured by showing the questionnaire to experts i.e., those working in the field of community pharmacy practice and experience of questionnaire design. Construct validity was not specifically tested in this study, due to the lack of existing measures of a similar construct. A questionnaire with good construct validity should correlate highly with tests that look at a similar construct, but it should not correlate with a test measuring an unrelated topic [31] Since this is the first questionnaire of its nature, no similar constructs exist for comparison. However, each of the sub-scales was highly correlated with each other, in the expected direction (i.e., all statistically significant strong positive correlations) which provides good initial support for its construct validity. The *PubPharmQ* findings relating to the awareness of the role of community pharmacy in public health, their expertise for this role, views about privacy, and the relationships in place were also largely supported by previous qualitative and quantitative research in this area [6,7,8,9,10,11,12,13,14,15,16,17,18,19,20,21,44,45,46,47] Predictive validity is the ability of a questionnaire to predict an outcome variable based on the correlation between the scales and a future outcome. Testing predictive validity was beyond the scope of this study; however, it is expected that higher scores on the four sub-scales of the *PubPharmQ* should predict more frequent pharmacy use for public-health services. A longitudinal study design is needed to explore this.

### 4.2. Reliability

Reliability is essential for the replicability of data. Cronbach’s alpha analyses showed that all four sub-scales of the *PubPharmQ* had excellent internal consistency indicating that the questionnaire has acceptable internal reliability. Other measures of reliability such as test-retest reliability [48] (which measures an individual’s scores across two tests at different times, where scores should remain largely the same to have acceptable test-retest reliability), were not explored. Future work should focus on expanding the testing of the questionnaire for reliability [49].

### 4.3. Strengths and Limitations

This study’s most notable strength is the robust mixed-methods approach undertaken to the development of the questionnaire. Conducting additional interviews to augment the focus group data previously gathered from our earlier study (where all focus group participants were all White-Caucasian ethnicity from North Wales) [16] enabled the capture of the views from other ethnic groups, as well as more young people (who tend to be healthy and are not generally users of community pharmacy services) and participants from a geographic location outside of Wales. The recruitment strategy for this study was successful in gaining responses from non-pharmacy users who can be more challenging to reach than regular pharmacy users who can be surveyed as part of a service evaluation. Two thirds of our sample did not visit the pharmacy regularly enough to pick up a prescription on a monthly or bi-monthly basis, and therefore would not be exposed to the full range of services that pharmacies can provide. This was achieved due to the high proportion of young adults (who may not traditionally use pharmacy services frequently) who responded to the questionnaire. It should be noted that although the age range of respondents was wide, the study sample was predominantly young adults, and as such the findings may not be representative of the wider population in terms of age distribution. Furthermore, while the higher proportion of female respondents to male participants in the sample was reflective of the population who most frequently use a community pharmacy [50] these findings may not be generalizable to male non-pharmacy users. Most participants who responded to the questionnaire were White, and therefore any potential ethnic and cultural differences in the public’s views about public-health roles in community pharmacy would not have been captured in this study. Similarly, most respondents were from either England or Wales with too few data from Scotland or Northern Ireland to identify any regional differences for the devolved nations and their different respective contractual frameworks. Socio-economic influences were not explored in this study, and this is a factor that could influence not only engagement with the research but also interpretation of the study findings.

Finally, one aspect that was not included in the questionnaire relates to public perceptions of pharmacies being easy to access, due to the lack of need for an appointment, weekend opening, and longer opening hours. The addition of items such as “It is easier to access my community pharmacy than my GP” and “It is easy to access support from my community pharmacy”, could provide useful insight into the importance of this as a facilitator use of public-health services. However, the issue of convenience has been widely reported in the literature and is a well-known and established enabler for public access to pharmacy services [8,9,18,45,51,52]. The *PubPharmQ* focuses on other determinants that are modifiable (i.e., level of privacy, awareness of role, relationship with pharmacy) and can be addressed as part of an intervention to promote awareness of pharmacy expertise for delivering public-health services.

### 4.4. Discussion of Findings

Most participants did not use the pharmacy regularly, indicating that they were not taking prescribed medication. Nevertheless, participants reported using mostly large multiples for their public-health needs (over half the sample) whereas these were the least likely to be used for prescription services (less than eight percent). This may be due to large multiples being better equipped to advertise their services with better financial resources, distribution lists, and access to TV advertising. This study was conducted in the early summer of 2021 while the COVID-19 pandemic was still prevalent in the UK. Public views towards community pharmacy may have changed since March 2020, because of their experiences of being able to leverage the accessible, convenient locations for key public-health services at a time when access to other NHS providers was restricted [29].

The questionnaire findings showed that older people reported stronger concerns about the need for privacy than young adults. The cross-sectional design of this study does not allow interpretation of the direction of this relationship. Older people may have had more experience dealing with various pharmacy staff members to discuss their medicines and other health-related needs over their lifetime and, as such, may have encountered some poor experiences with regard to having the appropriate level of privacy in place. Alternatively, this could be linked to older people who have more complex health needs that require a more in-depth conversation with pharmacy staff which they do not wish to be overheard. A Canadian study [45] found that older adults expressed greater awareness and more frequent use of community pharmacy services with multiple experiences associated with their care. Young adults reported having limited exposure to pharmacists and community pharmacy services, and some had never personally accessed the care of a pharmacist [45]. Their needs were expressed in terms of issues relevant to their stage in life, for example, services related to mental health and sexual and reproductive health, but developing a relationship with the pharmacist was as important as other factors, such as access, convenience, and location or type of pharmacy.

The issue of privacy is consistently reported in the literature as a barrier to engagement with CPs [6,7,8,9,10,11,12,13,14,15,16,17,18,19,20,21,44,45,46,47,50,51,52]. In this study, independent pharmacies were viewed most favorably, whereas supermarket pharmacies were rated poorly for privacy. However, over half of the participants had no experience of using a private consultation room within a pharmacy, and therefore may not be aware of their availability in all pharmacies, regardless of the type. Smaller, independent pharmacies may have fewer other people around (i.e., less footfall/”busyness”) which permits a more private conversation without the need to use a consultation room. Saramunee and colleagues [17] did not find any significant difference between age groups and a desire for a private room, but older people (>65 years) were statistically more likely than other age groups to trust a pharmacist and pharmacy staff to keep personal information confidential. Three quarters of the sample had experience of using a pharmacy regularly (at least once every 2–3 months). In contrast, younger participants (with less experience of using pharmacy) in the current study, reported a better perceived relationship with the community pharmacy team. These differences merit further exploration of the perceptions of community pharmacy, by users and non-pharmacy users, within different age categories, particularly concerning privacy and developing a good therapeutic relationship with the pharmacy. For example, certain demographic characteristics may influence the negative correlation between age and ‘Relationship’ with the pharmacy. While the primary goal of this study was to develop a robust questionnaire and not necessarily to focus on the results of the *PubPharmQ*, these issues merit further investigation to test these hypotheses.

### 4.5. Implications for Practice

Overall, our findings offer further support for the need to enhance public-health service utilization in community pharmacies in the UK by addressing the general public’s perspectives [46]. There is a need for better awareness of what services are provided in community pharmacies and to make sure that there is equity of access across all geographic locations. It is important to note that much of the previously published research [12,17,20,42] was conducted in England. To establish the best methods for the promotion of public-health services across the UK, we need to address differences in service provision and public perceptions across the devolved nations. The new community pharmacy contractual framework in Wales [5] makes an important step-change towards achieving this since community pharmacies must now either provide all the services available or none. Previously, contractors had the option of selecting individual services to offer from their pharmacy which was a source of confusion for members of the public. Our study supports the necessity of providing the same level of services from all community pharmacies to be able to develop consistent, effective messaging to optimize public awareness and engagement with public-health community pharmacy services.

### 4.6. Future Research

Further work is needed to continue the psychometric testing of the *PubPharmQ*, to determine its utility for predicting pharmacy use, and to discriminate between pharmacy and non-pharmacy users for public-health services [53]. Future research should also focus on reaching under-represented ethnic groups as well as targeted research in rural and urban communities or disadvantaged areas of the UK where public health needs are greatest [54]. Since data collected within community pharmacies are biased toward those who are already aware of the services on offer, places of worship, public libraries, or a trusted community individual could be used as a link to recruit members of the public from specific communities [55]. Finally, when considering the sample’s age range, considerations could be made for future iterations of the questionnaire in paper format (e.g., postal surveys) since older people might struggle to access the questionnaire online or may not use social media [56]. Obtaining the distribution of responders from the different types of social media platforms (i.e., Twitter, Instagram, LinkedIn) in future studies would also be beneficial since the audiences for each one can be very different.

## 5. Conclusions

The *PubPharmQ* provides a novel, robust, structured questionnaire to measure the public’s perceptions of community pharmacy’s role in public health. Based on these findings, considerable effort is needed to increase public awareness and address their concerns if strategic plans to utilize community pharmacies to engage in the delivery of public-health policy are to be successful. There is a need to promote the public-health services offered by community pharmacies in the UK and to encourage the public to make use of these roles. Educating the public about the pharmacy team’s qualifications, capabilities, and expertise for these roles will go some way toward achieving this. Furthermore, creating more private areas in the pharmacy and better use of consultation rooms has the potential to build the public’s trust in community pharmacy staff, thus fostering a better professional relationship. These findings may also be relevant to other countries outside of the UK where community pharmacies are offering these less traditional non-medication-related services.

## Figures and Tables

**Table 1:** Comparison of themes and sub-themes from interview and focus group data.

Interview Themes	Interview Sub-themes	Focus Group Themes	Focus Group Sub-Themes	Key Areas of Theme Alignment
1. Perceived role of role of CP	1.1 Medicine dispensing 1.2 Low-priority advice 1.3 Public health	Theme 1—Community pharmacist role Theme 3—Reason for visiting	1.1 Dispensing 1.2 Prescription medicine query/advise 1.3 Purchased medicine query/advice 1.4 Healthy living query/advice 1.5 Dietary query/advice 1.6 Minor ailment query/advice 1.7 Chronic condition management 3.1 OTC purchase 3.2 Toiletries purchase 3.3 Other products	Role in Public Health Reasons for Visiting
2. Perceived professional capability	2.1 Confusion regarding qualifications 2.2 Access to patient data 2.3 Knowledge of non-medical products	Theme 2—Professionalism of pharmacist	2.1 Role as part of NHS 2.2 Professional behavior 2.3 Professional knowledge 2.6 Professionalism of staff	Relationship Communication
3. Lack of established connection with a pharmacy	3.1 Fear of going to CP 3.2 Therapeutic relationship	Theme 2—Professionalism of pharmacist	2.4 Interprofessional relationships 2.5 Relationship with the public/patient	Relationship Communication
4 Privacy concerns	No sub-themes	Theme 2—Professionalism of pharmacist	2.7 Privacy	Privacy
5. Public awareness	5.1 Lack of awareness/under-utilization 5.2 Self-awareness 5.3 Using CP for specific problems	Theme 5—Accessibility	5.1 Convenience 5.2 Location	No alignment
6. Perception of the type of pharmacy	6.1 Professionalism 6.2 Specialism	Theme 4—Commercialism	4.1 Generic medication 4.2 Large multiples 4.3 Supermarket pharmacies 4.4 Small pharmacies	Reasons for Visiting

**Table 2:** Demographic characteristics of questionnaire respondents (*n* = 306).

Gender (Self-Identified as)	*Male*	67 (21.9%)
	*Female*	235 (76.8%)
	*Other*	2 (0.7%)
Age (In years)	*Range*	18 to 84
	*Mean (SD)*	34.5 (15.09)
	*Mode*	22
Nation of the UK	*England*	187 (61.1%)
	*Wales*	105 (34.3%)
	*Scotland*	11 (3.6%)
	*Northern Ireland*	1 (0.3%)
Ethnicity	*White*	263 (85.6%)
	*Mixed*	8 (2.6%)
	*Asian*	23 (7.5%)
	*Black*	7 (2.3%)
	*Other*	5 (1.65)
Employment	*Full-time*	87 (28.4%)
	*Part-time*	31 (10.1%)
	*Student (full-time)*	132 (43.1%)
	*Student (part-time)*	3 (1%)
	*Retired*	28 (9.2%)
	*Self-employed*	16 (5.2%)
	*Unemployed*	9 (2.9%)
Repeat Prescriptions	*Yes*	152 (49.7%)
	*No*	152 (49.7%)
	*Unsure*	2 (0.7%)

**Table 3:** Frequency of pharmacy visits in the last year for any reason (*n* = 306).

Number of Pharmacy Visits	Number of Participants *n* (%)
None	39 (12.7)
1–2	86 (28.1)
3–5	62 (20.3)
6–8	37 (12.1)
9–12	39 (12.7)
13–18	26 (8.5)
19–24	2 (0.7)
25+	15 (4.9)

**Table 4:** Type of pharmacy used in the last year for prescription and public-health needs (*n* = 306).

Type of Pharmacy	Prescription Needs *n* (%)	Type of Pharmacy	Public-Health Needs *n* (%) *
Small Multiple	104 (34)	Large Multiple	158 (51.6)
Supermarket	88 (28.8)	Independent	132 (43.1)
None used	54 (17.6)	Small Multiple	109 (35.6)
Independent	39 (12.7)	Supermarket	77 (25.2)
Large Multiple	21 (6.9)	None used	72 (23.5)

* For prescription needs, participants were asked to select one type of pharmacy; for public-health needs, participants could select multiple responses. Therefore, the total is over 100% due to this.

**Table 5:** Preferred type of community pharmacy for public-health issues, professionalism, comfort, and privacy (*n* = 306).

Type of Pharmacy	*n* (% *)				
	Preferred Pharmacy for Public-Health Support	Most Professional	Most Comfortable Attending	Most Concerned with Public-Health Matters	Most Privacy
Independent	124 (40.5)	104 (34)	116 (37.9)	77 (25.2)	112 (36.6)
Small Multiple	115 (37.6)	88 (28.8)	101 (33)	83 (27.1)	87 (28.4)
Large Multiple	130 (42.4)	97 (31.7)	122 (39.9)	81 (26.5)	77 (25.2)
Supermarket	43 (14.1)	21 (6.9)	35 (11.4)	25 (8.2)	19 (6.2)
No preference	90 (29.4)	95 (31)	92 (30.1)	92 (30.1)	43 (14.1)
Not sure	23 (7.5)	35 (11.4)	9 (2.9)	81 (26.5)	85 (27.8)

* Over 100% due to multiple response questions.

**Table 6:** Factors retained and removed from the Public Perceptions of Community Pharmacy’s Role in Public-Health Questionnaire (Version 2).

Scale Name/Scoring	Factors Retained/Scale Items	Factors Removed/Scale Items
*Expertise* 3 items; Min score = 3; Max Score = 15; Midpoint Score = 9	My local pharmacy team is qualified to provide me with public-health advice Community pharmacy staff have a lot of expertise in dealing with public health Community pharmacy staff have enough knowledge of my personal needs to advise me on public-health issues	I think of my local pharmacy team as medicine experts, not public-health experts Community pharmacy staff have a lot of expertise in dealing with public health I would prefer to have public-health advice from my pharmacy team than my GP
*Role in Public Health* 4 items; Min score = 4; Max Score = 20; Midpoint Score = 12	Community pharmacies do not play an important role in public health (*Score reversed*) Community pharmacies should focus only on providing medicines to the community (*Score reversed*) I do not think that my community pharmacy team would be able to help me quit smoking (*Score reversed*) My community pharmacy can only help me with a minor health issue, not public-health issues (*Score reversed*)	I visit my pharmacy to buy non-medical items only (e.g., toiletries, make-up) I can think of times when I have gone to my GP when I could have gone to my community pharmacy My local pharmacy is the best place to seek public-health advice I have a good understanding of the public-health services my community pharmacy provides The government should promote public-health services which are community pharmacy-based I have seen public-health services being promoted in my local pharmacy I feel like I can visit my local pharmacy for public-health issues I only visit my local pharmacy when I need medicine I visit my community pharmacy when I need advice for a specific condition (e.g., a skin condition)
*Relationship* 8 items; Min score = 8; Max Score = 40; Midpoint Score = 24	When I visit my local pharmacy, my needs are satisfied I have a good relationship with the community pharmacy team The community pharmacy team knows me on a first-name basis I feel nervous talking to community pharmacy staff about public-health issues (*Score reversed*) I can talk openly to the local pharmacy staff about my health I trust the local pharmacy staff I find it difficult to open up to the local pharmacy team about public-health issues (*Score reversed*) The local pharmacy team comes across as being professional	I would feel more comfortable going to the local pharmacy if I had a good relationship with the pharmacy team I would feel more comfortable if the community pharmacy team knew me on a first-name basis I feel that I would be able to talk openly to my local pharmacy team about my health I feel confident that the local pharmacy team would be professional in dealing with my public-health issue I feel nervous about the thought of talking to community pharmacy staff about public-health issues
*Privacy* 3 items; Min score = 3; Max Score = 15; Midpoint Score = 9	I have enough privacy when I go to the local pharmacy I worry that I can be overheard when I am in the local pharmacy (*Score reversed*) I feel comfortable going to the community pharmacy team to ask about sensitive health issues (e.g., sexual health)	Privacy is important to me when deciding if I visit the community pharmacy I do not trust the community pharmacy team with information about my health I trust the community pharmacy team as much as I trust the staff in my GP surgery I would be happy to use the private consultation rooms in the community pharmacy

**Table 7:** Scale Descriptions, Descriptive Statistics, and Cronbach’s Alpha (18-item scale) (*n* = 306).

Scale Description Scale Name—Number of Items Direction for Interpretation of Scoring	Range (Minimum–Maximum Scale Score)	Mean Scale Score	Standard Deviation	Cronbach’s Alpha
Community pharmacy team’s expertise on public health (*Expertise* Scale—3 items) High score = strong appreciation of CPs’ expertise in public-health matters	3–15	6.74	2.165	0.815
Community pharmacy’s role in public health (*Role in Public Health* Scale—4 items) High score = strong acceptance of CP’s role in delivering public-health services	4–20	9.19	2.981	0.745
Relationship with the community pharmacy team (*Relationship* Scale—8 items) High score = strong perceived relationship with the CP team	8–40	21.09	5.720	0.862
Privacy in the local pharmacy (*Privacy* Scale—3 items) High score = strong perception that CPs offer sufficient privacy	3–15	9.65	2.859	0.770

**Table 8:** Relationships (Pearson Correlations) between Scales.

		*Privacy*	*Relationship*		*Expertise in Public Health*
*Privacy*	Pearson Correlation r= Sig (2-Tailed) *N*=	1 - 306			
*Relationship*	Pearson Correlation r= Sig (2-Tailed) *N*=	0.598 0.000 306	1 - 306		
*Role in Public Health*	Pearson Correlation r= Sig (2-Tailed) *N*=	0.357 0.000 306	0.423 0.000 306	1 - 306	
*Expertise*	Pearson Correlation r= Sig (2-Tailed) *N*=	0.411 0.000 306	0.553 0.000 306	0.510 0.000 306	1 - 306

## Data Availability

The data presented in this study are available on request from the corresponding author. The data are not publicly available due to ethical restrictions.

## Supplementary Materials

The following supporting information can be downloaded at: https://www.mdpi.com/article/10.3390/pharmacy11050141/s1, Supplementary Material S1: Semi-structured interview schedule; Supplementary Material S2: Stage 1—Thematic analysis of interview data, Supplementary Material S3: Stage 2—Questionnaire; Supplementary Material S4; Stage 2—Exploratory Factor analysis results for questionnaire Version 1, Tables S1–S3; Supplementary Material S5: Stage 2—Total scale scores and frequency distributions, Figures S2–S5.
