# Supplementary material for: Development of a Questionnaire to Measure Public Perceptions of the Role of Community Pharmacy in Public Health (PubPharmQ)
Source: Pharmacy (Basel). 2023 Sep 8;11(5):141. doi: 10.3390/pharmacy11050141 (PMC10514790; doi:10.3390/pharmacy11050141)
Supplement: Supplementary file 1 [file pharmacy-11-00141-s001.zip › pharmacy-2556654-supplementary.pdf]

Supplementary Material S1

Stage 1 – Semi-Structured Interview Schedule

What do you think the main role of the CP is?

- Their main purpose

Can you tell me how would you feel about going to the CP for advice about a health problem?

Why would you use the CP?

- Services
- Products
- Any reason you would not use the CP?

How would you feel about asking the CP for a more intimate health matter?

- Use of consultation rooms

What do you think the differences are (if any) in pharmacists working in a supermarket pharmacy or a local pharmacy?

Does the fact that CPs advertise non-medical products like diet products affect how you feel about the CP?

- Prefer to buy diet products or ask for advice?

Can you tell me your thoughts on the pharmacy offering public health services such as blood pressure checks?

- Awareness?

How does the way that the CP confirms your identity when collecting a prescription make you feel?

- Confirm name and address

Supplementary Material S2

Stage 1 – Thematic Analysis of Interview Data

Thematic analysis of the interview data identified six themes (five emerged from the data and one as a result of direct questioning about type of community pharmacy) These were: *Perceived role, Public awareness, Perceived capability, Privacy concerns, Lack of connection and Type of pharmacy*. This is represented as a thematic map in Figure S1. A description of each theme is presented below with illustrative quotes to represent the theme or any sub-themes identified. Each participant was allocated a pseudonym to maintain anonymity.

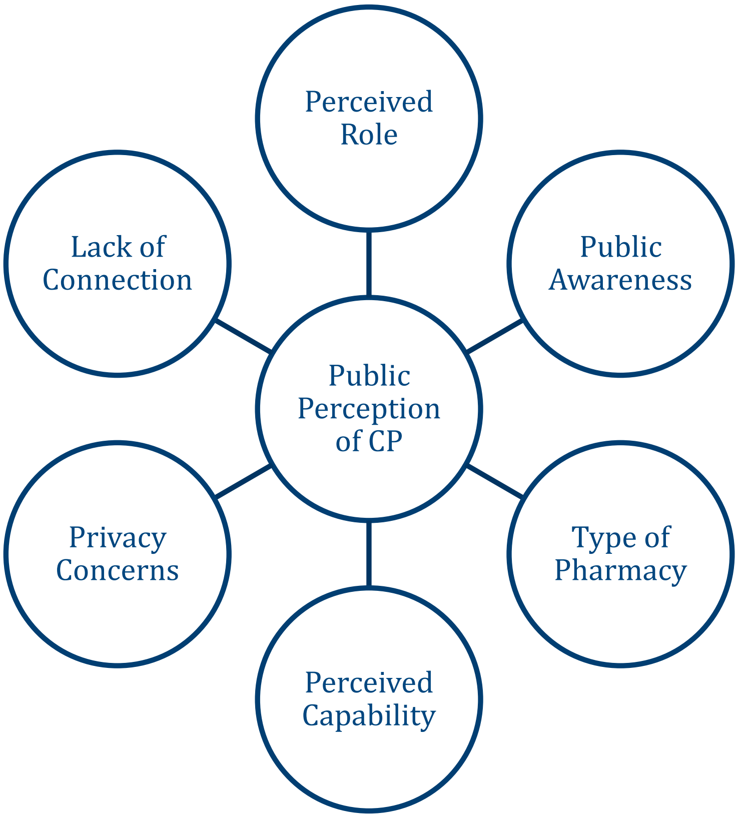

Figure S1: Thematic map of interview data

Theme 1: Perceived Role

This theme relates to the perceived role of the community pharmacy. Three sub-themes emerged in relation to this, which were *Medicine dispensing, Low priority advice* and *Public health roles*. Participants recognised that the CPs' roles expand beyond that of dispensing medication. Many knew of alternative services that the CP provides, such as offering health advice and raising awareness of public health issues among the wider community. Participants reported using the CP for non-urgent, less serious matters that may not require a visit to the GP.

> *"I do see them as um (3) a point of contact for non-urrrgent medical advice so something that I might be concerned about, but I probably don't need to see my doctor about" (Miriam, page 29, lines 3-6)*

The CP was highlighted as having the potential to be critical in promoting public health services and improving the overall health of the community.

> *"I think that that it it's just a way of raising awareness of the fact that if they don't care for their diet this is what's going to lead to more and more issues" (Yasmin, page 11, lines 162-164)*

Theme 2: Perceived Professional Capability

This overarching theme represents participants' perceptions of the professional capability of community pharmacy staff which were related to three sub-themes. These were, *Confusion regarding qualifications, Access to patient data* and *Knowledge of non-medical products.* There appeared to be some confusion around pharmacists' knowledge and training in comparison to that of General Practitioners (GPs). It is recognised that pharmacists are knowledgeable, but there was a perception that community pharmacists have less clinical knowledge than other healthcare professionals such as hospital pharmacists and GPs. Furthermore, supermarket pharmacies were viewed to be less knowledgeable, less professional and less specialised. This could potentially discourage individuals from using CPs as they may question whether they will receive the same level of care as they would from their GP.

> *"…pharmacists probably don't know a lot as say a doctor does" (Yasmin, page 11, lines 185-186)*

Access to medical notes was also highlighted as a potential barrier. Individuals felt that CPs had less knowledge and were less able to help due to not knowing their medical history. There was a consensus that pharmacists require this access and additional resources to build public trust in CPs and aim to increase utilisation of these services.

> *"…pharmacies - they don't have full records of a patient's history and I think that sometimes that can be an issue if the patient doesn't actually tell them what exactly is wrong with them…" (Yasmin, page 7, lines 32-34)*

Another concern that was thought to impact on trust and capability was the sale of non-medical items within pharmacies. Participants expressed the belief that pharmacists sell these items because they do not care, and that they do not have a good knowledge of what they are selling.

> *"…it might come across that they don't really care that much, they're just promoting stuff" (Padma, page 25, lines 47-49)*

Theme 3: Lack of Connection

Theme 3 captures participants' views about the perceived lack of connection of community pharmacy staff with the public. Two sub-themes were identified to represent this, which were, *Fear of going to CP* and *Therapeutic relationship* Participants noted that pharmacists see many people during the day and felt this may lead to a less personal service. People might be hesitant to use the CP and be more inclined to use the GP because they have already built a trusting relationship.

> *"…you don't really have a relationship with a pharmacist" (Prisha, page 20, line 35)*

> *"…with doctors there is a much more pronounced relationship" (Yasmin, page 9, lines 106-107)*

Furthermore, if individuals are reluctant to visit the CP due to feeling anxious or uncomfortable, this will make it difficult for relationships to form.

"…even when I've been ill just feel a bit awkward going I don't know why" (Padma, page 24, line 15)

Theme 4: Privacy Concerns

This theme represents concerns expressed about the lack of privacy in a community pharmacy. Concerns about privacy appeared to influence whether individuals felt they could trust their CP. Participants highlighted the importance of being able to discuss health concerns without others hearing and believed privacy would help them to view the CP as a trusted figure.

"…private and intimate matters like you know that no one is gonna like overhear it and stuff you're like kind of in trusted hands" (Prisha, page 21, lines 43-45)

Many participants were not aware of CP consultation rooms, but agreed that knowing these are available could be an important factor in deciding whether to go to the CP.

"…especially with the thing you said about the rooms I feel like yeah I definitely feel more comfortable being in that scenario instead of like going to a supermarket cos there's a lot of people around, it's like yeah" (Ben, page 18, lines 53-55)

Theme 5: Public Awareness

Theme 5 captures participants' perceived awareness of community pharmacy roles. Three sub-themes emerged relating to this, which were *Lack of awareness / underutilisation*, *Self-awareness* and *Using CP for specific problems*. Participants recognised that CPs may be underused due to a lack of public awareness of the services offered. They highlighted the need to promote CP services more widely through advertising and government initiatives to help alleviate pressures on GPs. It was suggested that people are motivated to use the CP when they are unwell but may be less aware of other services available such as blood pressure checks.

"…umm for example uh there's a lot of things that apparently, they can do that I didn't know about" (Anya, page 16, lines 112-113)

"…it doesn't make sense for me to go to a pharmacy unless I'm ill if I need anything like specific medicines from the pharmacy" (Grace, page 1, lines 24-26)

Theme 6: Type of Pharmacy

In response to the question relating to any perceived differences (if any) in pharmacists working in a supermarket pharmacy or a local pharmacy, two issues emerged in relation to *Professionalism* and *Specialism.* Concerns regarding supermarket pharmacy workers were raised, and they were perceived as less professional and less specialised than community pharmacy workers.

"yeah I think it's the environment that that's it it seems it seems far more general there um…" (*Miriam, page 30, lines 61-62)…. "far less um (3) professional I suppose" (Miriam, page 30, line 64)*

There was a perception that supermarket pharmacies are focused on medications whereas other types of pharmacies can offer more.

"…like supermarket pharmacies is just like focused on meds…" P(risha, page 21, line 59)

"…just went to like a normal supermarket they'll just tell you what they'll tell you what like medications and stuff" (Ben, 18, 48-5)

Supplementary Material S3

Stage 2 – Questionnaire

Establishing the General Public's Perceptions of Community Pharmacy

Community pharmacies are based in the heart of your local area. They are the most common way of accessing medications and health advice. Community Pharmacies can also offer support for public health matters such as providing healthy living advice, blood pressure checks, cholesterol checks, weight management, smoking cessation, and more. Public health is about promoting healthy living, preventing disease, and prolonging the lifespan. Community pharmacies can be independently owned pharmacies, small multiples such as Pearns Pharmacy (Wales), Allied Pharmacies (England), Right Medicine (Scotland), large multiples such as Boots or Lloyds, and pharmacies within supermarkets. Completing the questionnaire should take between 10 to 15 minutes on average.

<u>Part A: Some questions about you</u>

1. **Age (in years):**

2. **Gender Identity:**

   Female | Male | Other | Prefer not to say

3. **Your current location (first 4 digits of your postcode):**

4. **Ethnicity:**

   White (any white background) | Mixed/Multiple Ethnic Groups | Asian/Asian British) | Black/African/Caribbean/Black British | Other (please describe)

5. **Employment Status:**

   Full-Time Employed | Part-Time Employed | Full-Time student | Part-Time student | Self-Employed | Retired | Currently Unemployed

6. **Do you receive regular prescription medication from your GP?**

   Yes | No | Not sure

If yes, are these issued at regular intervals or as batches held at your pharmacy?

   Repeat | Batch | No | Not sure

7. **How many times in the last year did you visit a community pharmacy (for any reason)?**

   None | 1-2 | 3-5 | 6-8 | 9-12 | 13-18 | 19-24 | Over 25 times

8. **What type of pharmacy have you used the most in the past year to obtain prescription medicines (if at all)? Please tick one only**

   None (I have not had prescription medicines in the last year)

   Small Multiple (E.g. Sheppards, Rowlands Pharmacy)

   Large Multiple (E.g. Lloyds, Boots, Well)

   Supermarket Pharmacy (E.g., Asda, Sainsbury's, Tesco)

   Independent Pharmacy (Please Write Name Below)

9. **What type of pharmacy would you use for public health advice (if at all)? You can tick more than one option**

   None (I have not been to a pharmacy for public health advice)

   Small Multiple (E.g. Pearns, Rowlands Pharmacy)

   Large Multiple (E.g. Lloyds, Boots, Well)

   Supermarket Pharmacy (E.g., Asda, Sainsbury's, Tesco)

Independent Pharmacy (Please Write Name Below)

10. **Have you ever used a private consultation room when visiting a community pharmacy?**

    Yes | No | Not sure

If you have used a private consultation room in a community pharmacy, please can you provide further information on why you visited?

Part B: Your Views About Community Pharmacy and Public Health Roles

The following statements have been mentioned by others as part of previous research on community pharmacies. We would like to know what your personal views are on community pharmacy's role in public health. Please circle the option that applies most to you. There are no right or wrong answers, we are interested in your personal views.

1. Community Pharmacy's Role in Public Health

a. Community pharmacy staff have enough knowledge of my personal needs to advise me on public health issues.

Strongly Agree, Agree, Uncertain, Disagree, Strongly Disagree

b. My local pharmacy team is qualified to provide me with public health advice.

Strongly Agree, Agree, Uncertain, Disagree, Strongly Disagree

c. I think of my local pharmacy team as medicine experts, not public health experts.

Strongly Agree, Agree, Uncertain, Disagree, Strongly Disagree

d. Community pharmacy staff have a lot of expertise in dealing with public health.

Strongly Agree, Agree, Uncertain, Disagree, Strongly Disagree

e. I would prefer to have public health advice from my pharmacy team than my GP.

Strongly Agree, Agree, Uncertain, Disagree, Strongly Disagree

f. Community Pharmacies do not play an important role in public health.

Strongly Agree, Agree, Uncertain, Disagree, Strongly Disagree

g. Community pharmacies should focus only on providing medicines to the community.

Strongly Agree, Agree, Uncertain, Disagree, Strongly Disagree

h. I don't think that my Community Pharmacy team would be able to help people to quit smoking.

Strongly Agree, Agree, Uncertain, Disagree, Strongly Disagree

i. My Community Pharmacy team can only help me with a minor health issue, not public health issues.

Strongly Agree, Agree, Uncertain, Disagree, Strongly Disagree

j. My local pharmacy is the best place to get public health advice.

Strongly Agree, Agree, Uncertain, Disagree, Strongly Disagree

k. I have a good understanding of the public health services my community pharmacy provides.

Strongly Agree, Agree, Uncertain, Disagree, Strongly Disagree

l. The government should promote public health services which are community pharmacy based.

Strongly Agree, Agree, Uncertain, Disagree, Strongly Disagree

m. I have seen public health services being promoted in my local pharmacy.

Strongly Agree, Agree, Uncertain, Disagree, Strongly Disagree

Open Text Question: Do you have any further comments regarding the community pharmacy's role in offering public health advice?

2. <u>Reasons for Visiting my Community Pharmacy</u>

a. I feel like I can visit my local pharmacy for public health issues.

Strongly Agree, Agree, Neither Agree or Disagree, Disagree, Strongly Disagree

b. I only visit my local pharmacy when I need medicine.

Strongly Agree, Agree, Neither Agree or Disagree, Disagree, Strongly Disagree

c. I visit my Community Pharmacy when I need advice for a specific condition (e.g., a skin condition).

Strongly Agree, Agree, Neither Agree or Disagree, Disagree, Strongly Disagree

d. I visit my pharmacy to buy non-medical items only (e.g., toiletries, make-up).

Strongly Agree, Agree, Neither Agree or Disagree, Disagree, Strongly Disagree

e. I can think of times where I have gone to my GP when I could have gone to my Community Pharmacy.

Strongly Agree, Agree, Neither Agree or Disagree, Disagree, Strongly Disagree

f. When I visit my local pharmacy, my needs are satisfied.

Strongly Agree, Agree, Neither Agree or Disagree, Disagree, Strongly Disagree

Open text question: Do you have any further comments regarding your reasons for visiting (or not visiting) a community pharmacy?

3. <u>My Relationship with the Local Pharmacy</u>

a. I have a good relationship with the community pharmacy team.

Strongly Agree, Agree, Neither Agree or Disagree, Disagree, Strongly Disagree

b. The community pharmacy team knows me on a first-name basis.

Strongly Agree, Agree, Neither Agree or Disagree, Disagree, Strongly Disagree

c. I feel nervous talking to Community Pharmacy staff about public health issues

Strongly Agree, Agree, Neither Agree or Disagree, Disagree, Strongly Disagree

d. I can talk openly to the local pharmacy staff about my health.

Strongly Agree, Agree, Neither Agree or Disagree, Disagree, Strongly Disagree

e. I trust the local pharmacy staff.

Strongly Agree, Agree, Neither Agree or Disagree, Disagree, Strongly Disagree

f. I find it difficult to open up to the local pharmacy team about public health issues.

Strongly Agree, Agree, Neither Agree or Disagree, Disagree, Strongly Disagree

g. The pharmacy team comes across as being professional.

Strongly Agree, Agree, Neither Agree or Disagree, Disagree, Strongly Disagree

Open Text Question: Do you have any further comments regarding your relationship with the community pharmacy team?

4. Communicating with my Local Pharmacy Team

a. I would feel more comfortable going to the local pharmacy if I had a good relationship with the pharmacy team.

Strongly Agree, Agree, Neither Agree or Disagree, Disagree, Strongly Disagree

b. I would feel more comfortable if the community pharmacy team knew me on a first-name basis.

Strongly Agree, Agree, Neither Agree or Disagree, Disagree, Strongly Disagree

c. I feel nervous about the thought of talking to community pharmacy staff about public health issues.

Strongly Agree, Agree, Neither Agree or Disagree, Disagree, Strongly Disagree

d. I feel able to talk openly to my local pharmacy team about my health.

Strongly Agree, Agree, Neither Agree or Disagree, Disagree, Strongly Disagree

e. I feel confident that the local pharmacy team are professional in dealing with public health issues

Strongly Agree, Agree, Neither Agree or Disagree, Disagree, Strongly Disagree

Open Text Question: Do you have any further comments regarding communicating with the community pharmacy?

5. <u>Privacy in the Local Pharmacy</u>

a. I feel comfortable going to the Community Pharmacy team to ask about sensitive health issues (e.g., sexual health)

Strongly Agree, Agree, Neither Agree or Disagree, Disagree, Strongly Disagree

b. I have enough privacy when I go to the local Pharmacy.

Strongly Agree, Agree, Neither Agree or Disagree, Disagree, Strongly Disagree

c. I worry that I can be overheard when I am in the local pharmacy.

Strongly Agree, Agree, Neither Agree or Disagree, Disagree, Strongly Disagree

d. Privacy is important to me when deciding if I visit the Community Pharmacy.

Strongly Agree, Agree, Neither Agree or Disagree, Disagree, Strongly Disagree

e. I do **not** trust the Community Pharmacy team with information about my health.

Strongly Agree, Agree, Neither Agree or Disagree, Disagree, Strongly Disagree

f. I trust the Community Pharmacy team as much as I trust the staff in my GP surgery

Strongly Agree, Agree, Neither Agree or Disagree, Disagree, Strongly Disagree

g. I feel happy to use the private consultation rooms in the Community Pharmacy

Strongly Agree, Agree, Neither Agree or Disagree, Disagree, Strongly Disagree

Open Text Question: Do you have any further comments regarding your privacy in a community pharmacy?

6. Types of Pharmacy

a. Which type of pharmacy would you rather visit to get public health support? Please tick all that apply.

Independent | Small Multiple | Large Multiple | Supermarket | No Difference | Not Sure

b. Which type of pharmacy do you feel is the most professional? Please tick all that apply.

Independent | Small Multiple | Large Multiple | Supermarket | No Difference | Not Sure

c. Which type of pharmacy are you most comfortable going to? Please tick all that apply.

Independent | Small Multiple | Large Multiple | Supermarket | No Difference | Not Sure

d. Which type of pharmacy do you feel is most concerned with public health matters? Please tick all that apply.

Independent | Small Multiple | Large Multiple | Supermarket | No Difference | Not Sure

e. Which type of pharmacy do you feel has the most privacy? Please tick all that apply.

Independent | Small Multiple | Large Multiple | Supermarket | No Difference | Not Sure

Open Text Question: Do you have any further comments regarding the different types of pharmacy?

Thank You for Taking Part!

Supplementary Material S4

Stage 2 – Exploratory Factor Analysis Results for Questionnaire Version 1

Table S1 – Initial Factor Structure

Component	Initial Eigenvalues			Extraction Sums of Squared Loadings			Rotation Sums of Squared Loadings		
	Total	% of Variance	Cumulative %	Total	% of Variance	Cumulative %	Total	% of Variance	Cumulative %
1	11.717	30.833	30.833	11.717	30.833	30.833	5.281	13.896	13.896
2	2.707	7.123	37.956	2.707	7.123	37.956	4.623	12.165	26.061
3	2.034	5.354	43.310	2.034	5.354	43.310	2.812	7.401	33.463
4	1.956	5.147	48.457	1.956	5.147	48.457	2.722	7.163	40.626
5	1.448	3.810	52.267	1.448	3.810	52.267	2.550	6.710	47.337
6	1.421	3.740	56.007	1.421	3.740	56.007	2.044	5.380	52.717
7	1.141	3.003	59.011	1.141	3.003	59.011	1.879	4.944	57.660
8	1.109	2.918	61.929	1.109	2.918	61.929	1.622	4.269	61.929
9	0.998	2.626	64.555						
10	0.951	2.504	67.058						
11	0.922	2.427	69.485						
12	0.830	2.185	71.671						
13	0.806	2.121	73.791						
14	0.776	2.043	75.834						
15	0.727	1.912	77.747						
16	0.694	1.826	79.572						
17	0.626	1.648	81.221						
18	0.590	1.552	82.773						

19	0.514	1.354	84.126						
20	0.508	1.337	85.464						
21	0.486	1.278	86.742						
22	0.461	1.212	87.954						
23	0.426	1.122	89.076						
24	0.406	1.068	90.145						
25	0.383	1.008	91.153						
26	0.370	0.974	92.127						
27	0.335	0.882	93.009						
28	0.318	0.836	93.845						
29	0.301	0.791	94.637						
30	0.282	0.743	95.380						
31	0.275	0.725	96.105						
32	0.248	0.654	96.759						
33	0.230	0.605	97.364						
34	0.227	0.596	97.960						
35	0.219	0.577	98.538						
36	0.200	0.526	99.064						
37	0.188	0.494	99.558						
38	0.168	0.442	100.000						

Extraction Method: Principal Component Analysis.

Supplementary Material S4

Stage 2 – Simplified Rotated Factor-Loading Matrix for Questionnaire Version 2

Table S2 – Rotated Factor Matrix for the 18-item Questionnaire (Version 2)

	Factor 1 - Expertise	Factor 2 - Relationship	Factor 3 - Privacy	Factor 4 – Role
Community pharmacy staff have enough knowledge of my personal needs to advise me on public health issues.	**0.719**			
My local pharmacy team is qualified to provide me with public health advice.	**0.758**			
Community pharmacy staff have a lot of expertise in dealing with public health.	**0.675**			0.340
Community Pharmacies do not play an important role in public health.	0.376			**0.532**
Community pharmacies should focus only on providing medicines to the community.				**0.658**
I don’t think that my Community Pharmacy team would be able to help people to quit smoking.				**0.605**
My Community Pharmacy team can only help me with a minor health issue, not public health issues.				**0.665**
When I visit my local pharmacy, my needs are satisfied.	0.539	**0.408**		
I have a good relationship with the community pharmacy team.		**0.425**		
The community pharmacy team knows me on a first-name basis.		**0.332**		
I feel nervous talking to Community Pharmacy staff about public health issues		**0.807**		
I can talk openly to the Local Pharmacy staff about my Health	0.364	**0.686**		
I trust the Local Pharmacy staff	0.639	**0.407**		
I find it difficult to open up to the Local Pharmacy team about public health issues.		**0.732**		
The Local Pharmacy team comes across as being professional	0.641	**0.383**		

I feel comfortable going to the Community Pharmacy team to ask about sensitive health issues (e.g., sexual health).		0.387	**0.513**	
I have enough privacy when I go to the Local Pharmacy.			**0.709**	
I worry that I can be overheard when I am in the Local Pharmacy.			**0.744**	

Extraction Method: Principal Component Analysis. Rotation Method: Varimax

Supplementary Material S4

Stage 2 - Original (Version 1) and New Questionnaire (Version 2) Items Following Factor Analysis

Table S3: Original 38-item questionnaire and New 18-item questionnaire following Exploratory Factor Analysis

Original Questionnaire Section	Items in Original Section	New Scale Following Factor Analysis	Items in New Scale with Correlation Coefficient
**Section 1: Community Pharmacy's Role in Public Health**<br><br>13 items	Community pharmacy staff have enough knowledge of my personal needs to advise me on public health issues<br><br>My local pharmacy team is qualified to provide me with public health advice<br><br>I think of my local pharmacy team as medicine experts, not public health experts (Reversed)<br><br>Community pharmacy staff have a lot of expertise in dealing with public health<br><br>I would prefer to have public health advice from my pharmacy team than my GP<br><br>Community pharmacies do not play an important role in public health (Reversed)<br><br>Community pharmacies should focus only on providing medicines to the community (Reversed)<br><br>I don't think that my community pharmacy team would be able to help people quit smoking (Reversed)<br><br>My community pharmacy team can only help me with a minor health issue, not public health issues (Reversed)<br><br>My local pharmacy is the best place to get public health advice<br><br>I have a good understanding of the public health services my community pharmacy provides	**Community Pharmacy Team's Expertise in Public Health**<br><br>**Scale Name = Expertise**<br><br>3 items<br>Min score = 3<br>Max Score = 15<br>Midpoint Score = 9	Community pharmacy staff have enough knowledge of my personal needs to advise me on public health issues (0.719)<br><br>My local pharmacy team is qualified to provide me with public health advice (0.758)<br><br>Community pharmacy staff have a lot of expertise in dealing with public health (0.675)

	The government should promote public health services which are community pharmacy based<br><br>I have seen public health services being promoted in my local pharmacy		
**Section 2: Visiting the Community Pharmacy**<br><br>6 items	I feel like I can visit my local pharmacy for public health issues<br><br>I only visit my local pharmacy when I need medicine<br><br>I visit my community pharmacy when I need advice for a specific condition (e.g., a skin condition)<br><br>I only visit my pharmacy to buy non-medical items (e.g., toiletries, make-up)<br><br>I can think of times where I have gone to my GP when I could have gone to my community pharmacy<br><br>When I visit my local pharmacy, my needs are satisfied<br><br>Do you have any further comments regarding your reasons for visiting (or not visiting) a community pharmacy? (open text answer)	**Community Pharmacy's Role in Public Health**<br><br>**Scale Name = Role**<br><br>4 items<br>Min score = 4<br>Max Score = 20<br>Midpoint Score = 12	Community pharmacies do not play an important role in public health (0.532) (Likert scale score Reversed)<br><br>Community pharmacies should focus only on providing medicines to the community (0.658) (Likert scale score Reversed)<br><br>I don't think that my community pharmacy team would be able to help me quit smoking (0.605) (Likert scale score Reversed)<br><br>My community pharmacy can only help me with a minor health issue, not public health issues (0.665) (Likert scale score Reversed)
**Section 3: My Relationship with the Local Pharmacy**<br><br>7 items	I have a good relationship with the community pharmacy team<br><br>The community pharmacy team knows me on a first-name basis<br><br>I feel nervous talking to community pharmacy staff about public health issues (Reversed)<br><br>I can talk openly to the local pharmacy staff about public health issues<br><br>I trust the local pharmacy staff<br><br>I find it difficult to open up to the local pharmacy team about public health issues (reversed)<br><br>The local pharmacy team comes across as being professional	**Relationship with the Community Pharmacy Team**<br><br>**Scale Name = Relationship**<br><br>8 items<br>Min score = 8<br>Max Score = 40<br>Midpoint Score = 24	When I visit my local pharmacy, my needs are satisfied (0.408)<br><br>I have a good relationship with the community pharmacy team (0.425)<br><br>The community pharmacy team knows me on a first-name basis (0.332)<br><br>I feel nervous talking to community pharmacy staff about public health issues (0.807) (Likert scale score Reversed)<br><br>I can talk openly to the local pharmacy staff about my health (0.686)<br><br>I trust the local pharmacy staff (0.407)<br><br>I find it difficult to open up to the local pharmacy team about public

			health issues (0.732) (Likert scale score Reversed)<br><br>The local pharmacy team comes across as being professional (0.383)
**Section 4: Communicating with my Local Pharmacy Team**<br><br>5 items	I would feel more comfortable going to the local pharmacy if I had a good relationship with the pharmacy team<br><br>I would feel more comfortable if the community pharmacy team knew me on a first-name basis<br><br>I feel nervous about the thought of talking to community pharmacy staff about public health issues (Reversed)<br><br>I feel that I would be able to talk openly to my local pharmacy team about my health<br><br>I feel confident that the local pharmacy team would be professional in dealing with my public health issue	**Privacy in the Local Pharmacy**<br><br>**Scale name = Privacy**<br><br>3 items<br>Min score = 3<br>Max Score = 15<br>Midpoint Score = 9	I have enough privacy when I go to the local pharmacy (0.709)<br><br>I worry that I can be overheard when I am in the local pharmacy (0.744) (Likert scale score Reversed)<br><br>I feel comfortable going to the community pharmacy team to ask about sensitive health issues (e.g. sexual health) (0.513)
**Section 5: Privacy in the Local Pharmacy**<br><br>7 items	I feel comfortable going to the community pharmacy team to ask about sensitive health issues (e.g. sexual health)<br><br>I have enough privacy when I go to the local pharmacy<br><br>I worry that I can be overheard when I am in the local pharmacy (Reversed)<br><br>Privacy is important to me when deciding if I visit the community pharmacy<br><br>I do not trust the community pharmacy team with information about my health (Reversed)<br><br>I trust the community pharmacy team as much as I trust the staff in my GP surgery<br><br>I would feel happy to use the private consultation rooms in the community pharmacy	N/A	N/A
**Total 38 Items**		**Total 18 Items**	

Supplementary Material S5

Stage 2 - Total Scale Scores and Frequency Distributions –

Community Pharmacy team's expertise on public health had a maximum score of 15, therefore above the midpoint score of **9** indicates high agreement on community pharmacy's having expertise in public health. The mean score was 6.74 (SD=2.165), the median was 7, and only 7.8% scored above the midpoint indicating low appreciation for the expertise of CPs in public health (see Figure S2).

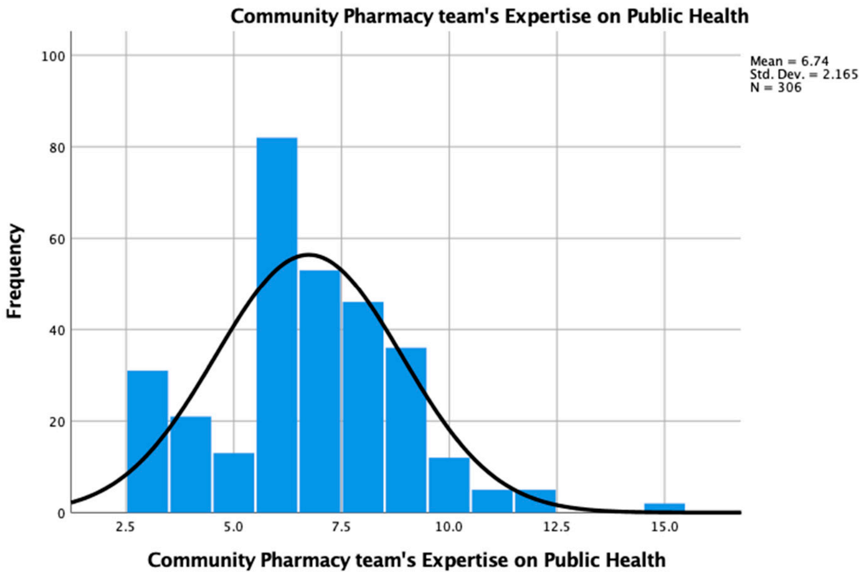

Figure S2 – Expertise scale scores (n=306)

Community Pharmacy's Role in Public Health had a maximum score of 20, therefore above the midpoint score of 12 indicates high agreement with the community pharmacy's role in public health. The mean score was 9.19 (SD=2.981), the median was 9, and only 12.7% scored over the midpoint indicating low appreciation of the community pharmacy team's role in public health (Figure S3).

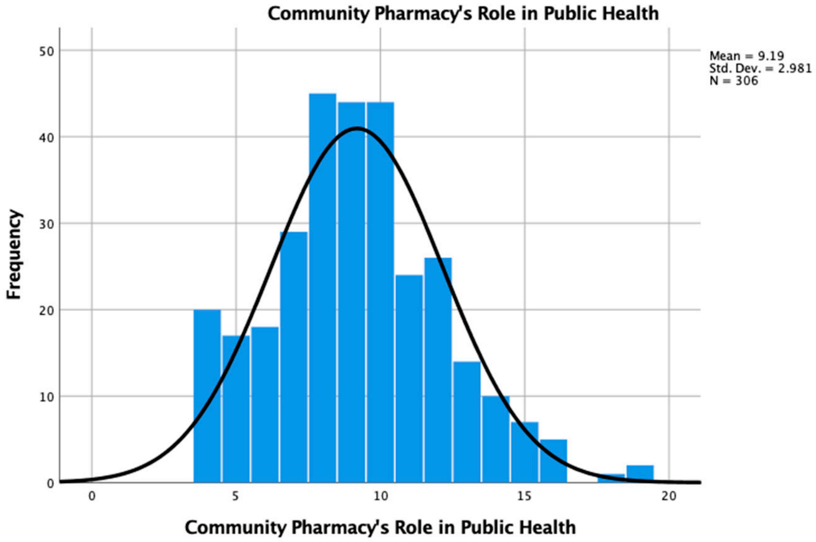

Figure S3 – Role in Public Health Scale Scores (n=306)

Relationship with the community pharmacy team had a maximum score of 40, and a midpoint score of 24. Above this score shows high agreement with a good relationship with the community pharmacy (Figure S4). The mean score was 21.9 (SD= 5.720), the median was 21, and 27.1% scored above the midpoint indicating a modest appreciation of the therapeutic relationship with CP staff.

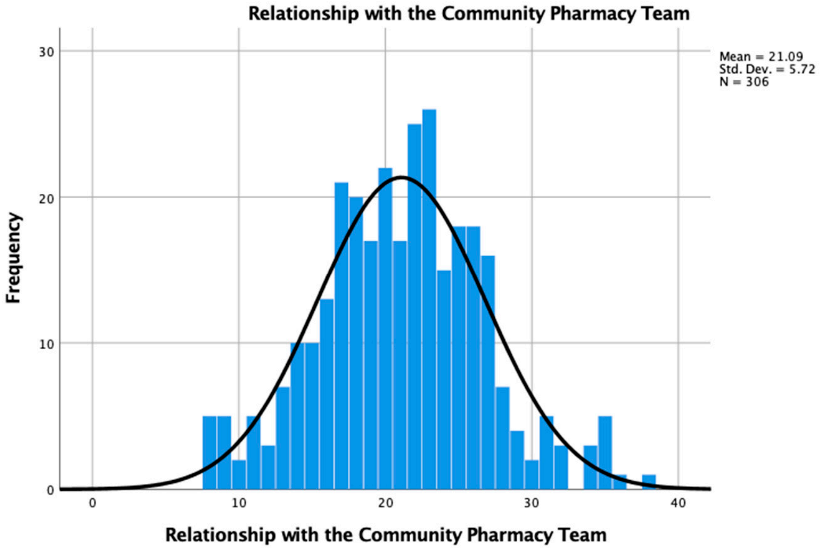

Figure S4 – Relationship Scale Scores (n=306)

Privacy in the local pharmacy has a maximum score of 15, therefore a midpoint score of above 9 indicates high agreement with privacy scale scores (Figure S5). The mean score was 9.65 (SD=2.859), the median was 10, and only 54.6% scored above the midpoint indicating that concerns over privacy is an issue for nearly half the participants.

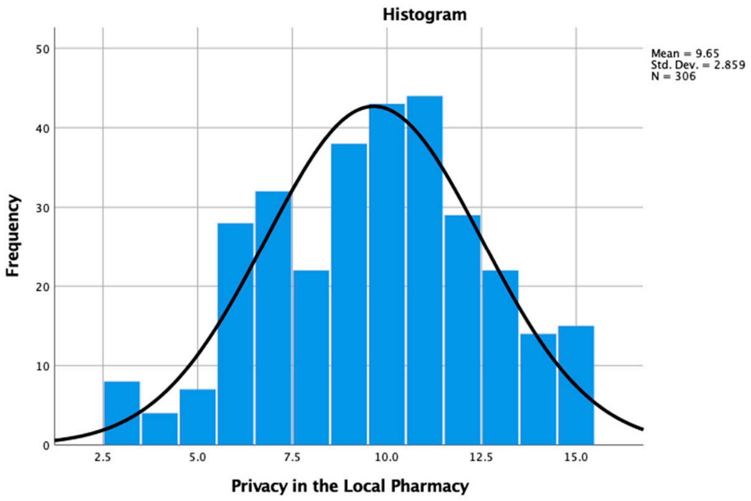

Figure S5 – Privacy scale scores (n=306)
